# Identification of a ferroptosis-related gene signature predictive model in colon cancer

**DOI:** 10.1186/s12957-021-02244-z

**Published:** 2021-04-29

**Authors:** Ye Wang, Heng-bo Xia, Zhang-ming Chen, Lei Meng, A-man Xu

**Affiliations:** Department of General Surgery, First Affiliated Hospital of Anhui Medical University, Hefei, 230022 Anhui Province People’s Republic of China

**Keywords:** TCGA, Ferroptosis-related genes, Colon cancer, Prognosis model

## Abstract

**Background:**

The prognosis of colon cancer (CC) is challenging to predict due to its highly heterogeneous nature. Ferroptosis, an iron-dependent form of cell death, has roles in various cancers; however, the correlation between ferroptosis-related genes (FRGs) and prognosis in CC remains unclear.

**Methods:**

The expression profiles of FRGs and relevant clinical information were retrieved from the Cancer Genome Atlas (TCGA) database. Cox regression analysis and the least absolute shrinkage and selection operator (LASSO) regression model were performed to build a prognostic model in TCGA cohort.

**Results:**

Ten FRGs, five of which had mutation rates ≥ 3%, were found to be related to the overall survival (OS) of patients with CC. Patients were divided into high- and low-risk groups based on the results of Cox regression and LASSO analysis. Patients in the low-risk group had a significantly longer survival time than patients in the high-risk group (*P* < 0.001). Enrichment analyses in different risk groups showed that the altered genes were associated with the extracellular matrix, fatty acid metabolism, and peroxisome. Age, risk score, T stage, N stage, and M stage were independent predictors of patient OS based on the results of Cox analysis. Finally, a nomogram was constructed to predict 1-, 3-, and 5-year OS of patients with CC based on the above five independent factors.

**Conclusion:**

A novel FRG model can be used for prognostic prediction in CC and may be helpful for individualized treatment.

**Supplementary Information:**

The online version contains supplementary material available at 10.1186/s12957-021-02244-z.

## Introduction

Colorectal cancer (CC) is the third most commonly diagnosed carcinoma and the second most common cause of cancer death worldwide. Indeed, the latest global cancer statistics has shown that CC still accounts for over 1.85 million new cases per year and an estimated 880,792 deaths per year (equating to 1 in every 12 deaths globally) [1]. Although continuous developments in early detection and treatment have led to a decline in the mortality and incidence of CC, 30–50% of patients present with metastasis or recurrence within 5 years after treatment [2, 3]. Meanwhile, it is still not possible to accurately predict the survival time of patients with CC, largely due to tumor heterogeneity caused by both genetic and environmental factors [4]. Therefore, a reliable prognosis assessment model is eagerly awaited to predict the prognosis of patients with CC and to optimize clinical treatment strategies. Furthermore, with the development of next-generation sequencing technology, the perception of the cancer molecular network and transcriptomic analysis can provide better technical support for prediction of CC prognosis [5, 6].

Iron is a necessary element for both humans and microorganisms. However, iron overload can harm cells through a variety of mechanisms, including the induction of cell death. Ferroptosis is an iron-dependent form of cell death caused by persistent membrane injury and continuous lipid peroxidation [7]. Ferroptosis is regulated by a variety of metabolism-related genes. The enzyme glutathione peroxidase 4 (GPX4) is one of the key regulators of ferroptosis, which protects cells by neutralizing lipid peroxides, and direct inhibition of GPX4 can trigger ferroptosis [8]. Numerous genes are known to influence cancer, especially aggressive malignancies by triggering ferroptosis [9, 10]. For example, *p53*, a key cancer-suppressor gene, can impede cancer development by inhibiting cystine uptake and sensitizing cells to ferroptosis [11]. Moreover, ferroptosis can affect chemosensitivity through important signaling pathways such as the β-catenin/Wnt signaling pathways [12]. In line with this, ferroptosis inducers are thought to represent a potential treatment strategy for therapy-resistant cancers [13]. Previous studies have shown that some genes such as *SLC7A11* and *ACADSB* can attenuate the proliferation of CC cells via triggering ferroptosis [14, 15]. In addition, some drugs have been shown to suppress CC by stimulating some level of ferroptosis [16]. However, whether the expression of ferroptosis-related genes (FRGs) is related to the prognosis of patients with CC remains unclear.

In this study, we explored the relationship between the expression of FRGs and OS in patients with CC. To this end, we built a nomogram model to predict the OS of patients with CC.

## Materials and methods

### Data acquisition

The RNA sequencing data and relevant clinical information of CC samples were downloaded from The Cancer Genome Atlas (TCGA) database (https://portal.gdc.cancer.gov). The dataset contained 41 adjacent normal samples and 473 tumor samples. Samples without complete survival and clinical data were removed, and samples from patients with an OS < 60 days were excluded. Finally, 353 samples with complete clinical stage data were included in the follow-up work. All processes relating to the selection and analysis of data are shown in Additional File 1. The gene expression was normalized using log2 (fragments per kilobase of exon model per million mapped fragments (FPKM) + 0.01). All data from TCGA are publicly available according to the TCGA data access policies. Thus, this study did not require Ethics Committee approval. A list of 259 FRGs was constructed using the ferroptosis database (FerrDb; http://www.zhounan.org/ferrdb) [17], a publicly available database of ferroptosis regulators, markers, and disease associations.

### Construction of a prognostic FRG signature

The Wilcoxon test was performed to determine the ferroptosis-related differentially expressed genes (DEGs) between adjacent normal tissues and tumor tissues with *P* < 0.05 and a false discovery rate (FDR) < 0.05 in the TCGA cohort. Univariate Cox analysis was used to screen out FRGs with prognostic value. The mutation rates of prognostic FRGs were analyzed by the cBioPortal for Cancer Genomics online website (https://www.cbioportal.org) [18]. Least absolute shrinkage and selection operator (LASSO) regression analysis, a common method of performing regression analysis with high dimensional factors, was used to construct a prognostic model to minimize the level of overfitting [19, 20]. Three-fold cross-validation was conducted to reduce the potential instability of the results, and the optimal tuning parameter λ was identified based on a 1-SE (standard error) standard. The “glmnet” R package was used for the LASSO analysis to select the variables in this study. The risk scores were analyzed on the basis of the expression level of each gene and its corresponding regression coefficients. The risk scores were calculated using the following formula: risk score = (gene expression level × corresponding coefficient). Patients with CC were divided into high- and low-risk groups according to the median risk score. The distributional difference of different groups was analyzed using t-distributed stochastic neighbor embedding (t-SNE) and principal component analysis (PCA) with the “Rtsne” and “stats” R packages. The Kaplan–Meier plot and log-rank test were used to evaluate survival differences between the high- and low-risk groups. Receiver operator characteristic curves (ROC) and area under the curves (AUC) were used to evaluate the availability of the prognosis model via the “survivalROC” R package. Decision-curve analysis (DCA) was applied to evaluate the clinical applicability of the constructed nomogram and to quantify the net improved benefits at various thresholds.

### Functional enrichment analysis

Gene ontology (GO) analysis and gene set enrichment analysis (GSEA) were performed to determine the biological functions of DEGs between the two groups. GO analysis was performed using the “org.Hs.eg.db” R package, with *P*-values and FDR values < 0.05. GSEA was used to analyze the enrichment of DEGs in Kyoto Encyclopedia of Genes and Genomes (KEGG) gene sets, with *P*-values < 0.05 and FDR values < 0.25.

### Statistical analysis

Categorical variables were presented as count (percentage) and analyzed by the chi-square test. Univariate and multivariate Cox analyses were performed to identify independent prognostic factors, which were used to build the nomogram model to predict the 1-, 3-, and 5-year OS rates [21]. The Harrell C statistic and the calibration plot were adopted to evaluate the discrimination and calibration, respectively. All statistical analyses and plots were performed in R version 3.6.2. *P-*values < 0.05 were considered significant.

## Results

### Identification of prognostic FRGs in the colon TCGA cohort

**Fig. 1:**
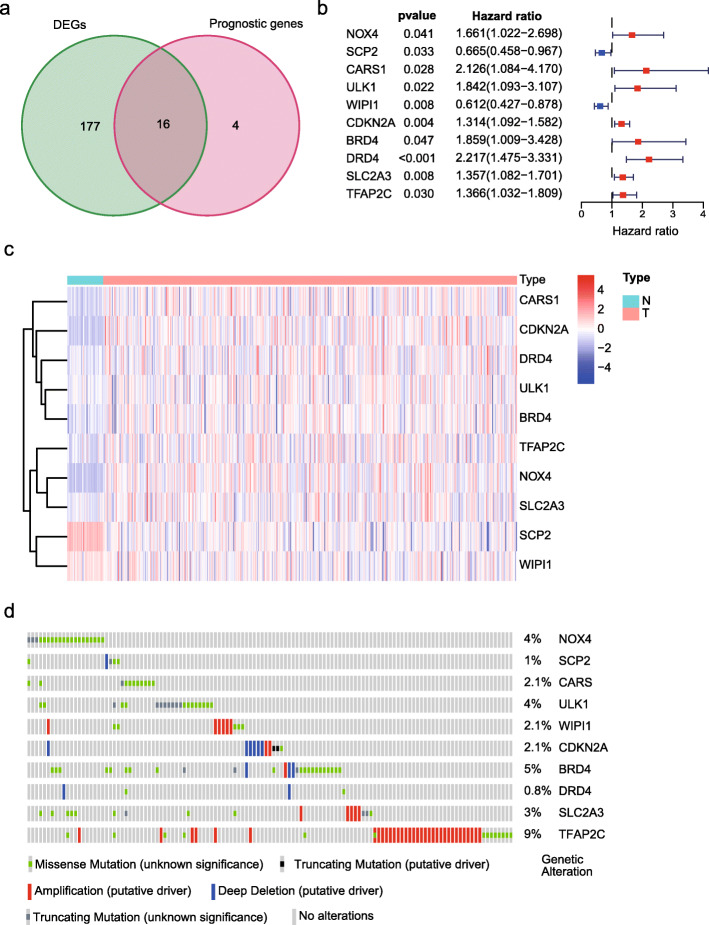
Identification of candidate FRGs in TCGA cohort. **a** Venn diagram to identify ferroptosis-related DEGs between tumor and adjacent normal tissue that were correlated with OS. **b** Forest plots showing the results of univariate Cox regression analysis between the expression of 10 candidate FRGs and OS. **c** Heat map showing the expression of 10 candidate FRGs in normal and tumor colon tissue. **d** Mutation information of prognosis-related FRGs; TFAP2C was the most frequently mutated gene, and five genes had a mutation rate ≥ 3%

Most of the FRGs were differentially expressed (FDR < 0.05) between adjacent normal tissues and tumor tissues, and 16 of the DEGs were related to OS (Fig. 1a). Five genes were downregulated in tumor samples, but the higher expression of these genes predicted poorer prognosis. Meanwhile, *MYB* was upregulated in tumor tissues, but its higher expression showed better prognosis. Therefore, these 6 genes were excluded from further study. The prognosis and differential expression of 10 FRGs are shown in Fig. 1a, b. Mutational information of these 10 genes showed that amplifications, deep deletion, and missense mutations were the most frequent mutation types (Fig. 1d). Five FRGs had mutation rates ≥ 3%, and *TFAP2C* had the highest mutation rate.

### Construction of a risk score model

**Fig. 2:**
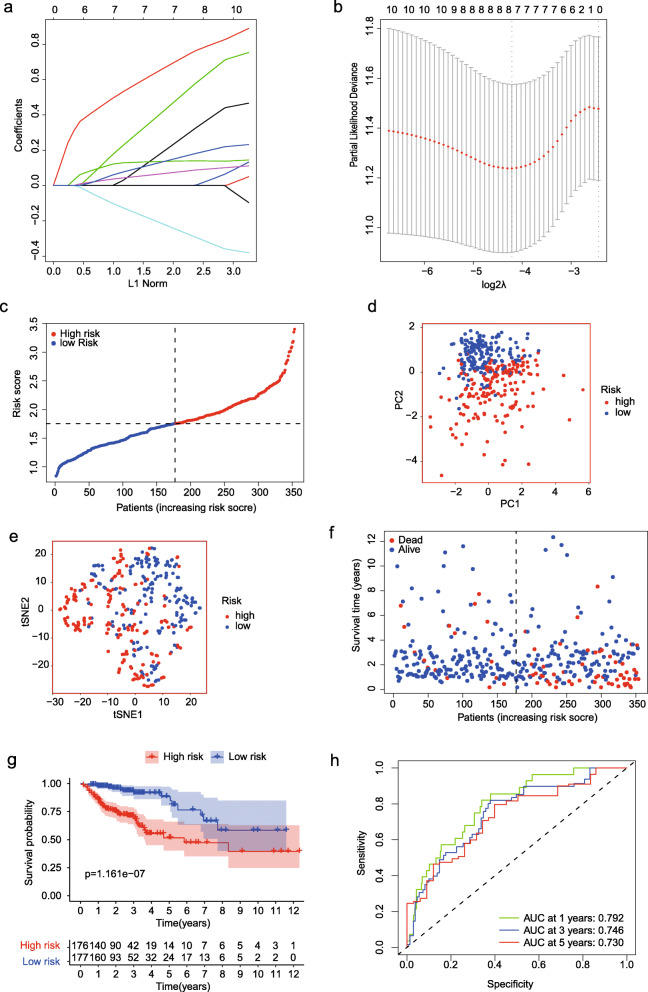
Establishment of a prognostic gene signature by LASSO regression analysis. **a** LASSO coefficient profiles of the 10 genes in colon cancer samples. **b** A coefficient profile plot was generated against the log (lambda) sequence. Selection of the optimal parameter (lambda) in the LASSO model for colon cancer. **c** The distribution and median value of the risk scores in the Cancer Genome Atlas (TCGA) cohort. **d** PCA plot of the TCGA cohort. **e** t-SNE analysis of the TCGA cohort. **f** OS status, OS, and risk score in the TCGA cohort. **g** Kaplan–Meier curves for the OS of patients in the high- and low-risk groups in the TCGA cohort. **h** AUC of time-dependent ROC curves verified the prognostic performance of the risk score in the TCGA cohort

After filtering out the genes without prognostic significance, LASSO regression analysis was used to develop a risk score by analyzing the expression level of the 10 FRGs mentioned above (Fig. 2a). Seven FRGs were determined on account of the optimal value of λ (Fig. 2b), and a risk formula was constructed with the expression levels of seven genes. The patients were divided into high- and low-risk groups (Fig. 2c), and PCA and t-SNE analysis demonstrated that the two groups of patients distributed in two different dimensions (Fig. 2d–e). As shown in Fig. 2f, patients in the high-risk group were more likely to die earlier than those in the low-risk group. Similarly, the Kaplan–Meier plot demonstrated that patients in the high-risk group had a worse prognosis than those in the low-risk group (Fig. 2g; *P* < 0.001). The ROC curve was used to evaluate the predictive effect of the risk score for OS, and the AUC was 0.792 at 1 year, 0.746 at 3 years, and 0.730 at 5 years (Fig. 2h).

### Functional analyses in different risk groups

**Fig. 3:**
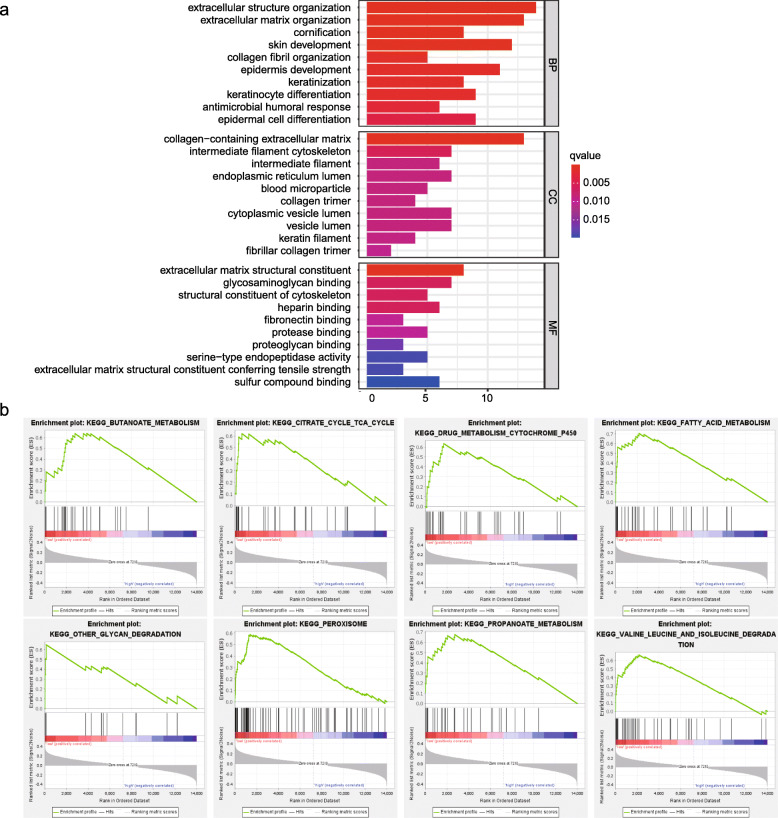
Functional enrichment analyses in the TCGA cohort. **a** Gene ontology (GO) analysis showing the biological processes, cellular components, and molecular functions’ enrichment of differentially expressed genes (DEGs) in two groups. **b** GSEA shows the most significant pathway enrichments of DEGs in the two groups

The DEGs between the two groups were used to analyze the relationship between biological functions and risk score. As expected, GO analysis demonstrated that the DEGs were mainly related to biological processes (BP), cellular components (CC), and molecular functions (MF) of the extracellular matrix (Fig. 3a). Furthermore, the altered genes were significantly enriched in eight KEGG pathways by GSEA, with *P* < 0.05 and FDR < 0.25 (Fig. 3b). We found that DEGs were significantly associated with the fatty acid metabolism and peroxisome (NES = 1.99, *P* ≤ 0.001). The detailed results of GO analysis and GSEA are shown in Additional Files 2 and 3.

### Clinical features and prognostic value of the risk score

**Fig. 4:**
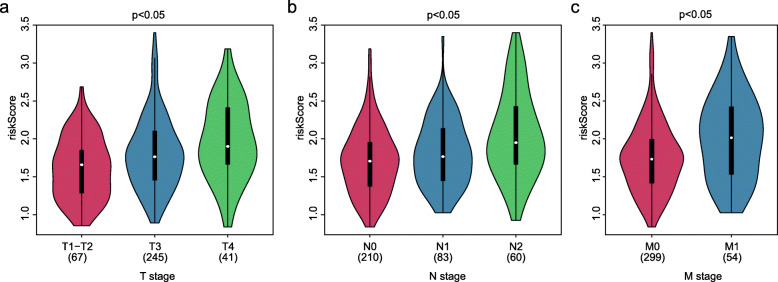
Association between TNM stage and risk score. Violin plots showing the risk score at different **a** tumor infiltration depths, **b** lymph node metastasis, and **c** distant metastasis

**Fig. 5:**
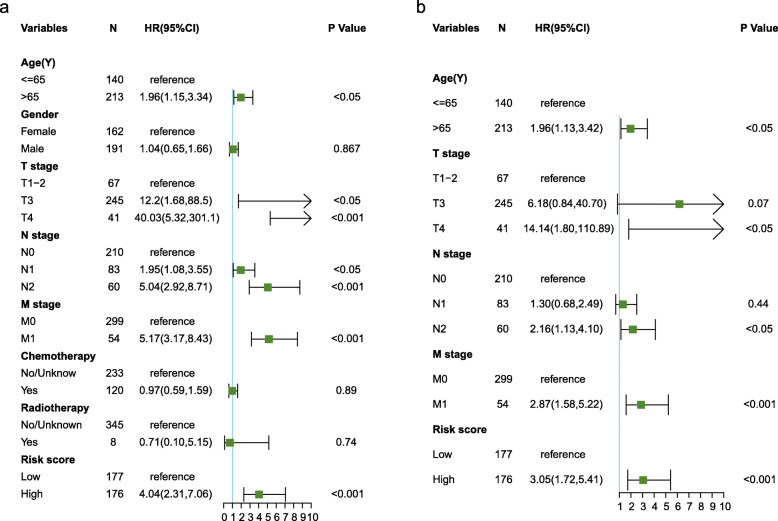
The prognostic value of clinical features and the risk score. **a** Univariate Cox regression analyses regarding OS in the TCGA cohort. **b** Multivariate Cox regression analyses regarding OS in the TCGA cohort

**Fig. 6:**
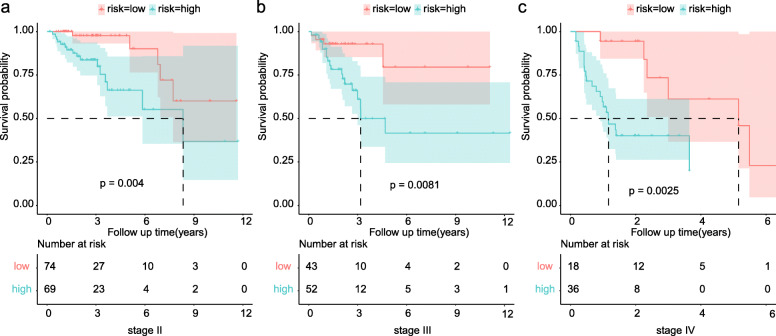
Impact of risk groups on prognosis by subgroup analysis. Survival differences between the high- and low-risk groups in stage II (**a**), stage III (**b**), and stage IV (**c**)

As shown in Table 1, the T stage, N stage, and M stage in the high-risk group were more advanced than those in the low-risk group. Furthermore, violin plots demonstrated that the calculated risk score was positively related to the tumor infiltration depth (Fig. 4a), lymph node metastasis (Fig. 4b), and distant metastasis (Fig. 4c). Otherwise, age, T stage, N stage, M stage, and risk score were independent risk factors for OS (Fig. 5a–b). The risk score was also significantly related to OS (univariate: hazard ratio [HR], 4.04; 95% CI, 2.31–7.06; *P* < 0.001; multivariate: HR, 3.05; 95% CI, 1.72–5.41; *P* < 0.001). In order to exclude the impact of tumor stages on the prognosis of the risk group, we analyzed the prognostic value of the risk score in groups with different AJCC stages. The Kaplan–Meier plots demonstrated that the prognosis of the high-risk group was worse than that of the low-risk group in stage II, stage III, and stage IV (Fig. 6a–c).

**Table 1:** Clinical characteristics of the colon patients between the high- and low-risk groups

Variables	Group		
	High risk	Low risk	***P*** value
**Age**			0.071
<65	61 (34.7%)	79 (44.6%)	
≥65	115 (65.3%)	98 (55.4%)	
**Gender**			0.221
Female	87 (49.4%)	75 (42.4%)	
Male	89 (50.6%)	102 (57.6%)	
**T stage**			0.002
T1-T2	22 (12.5%)	45 (25.4%)	
T3	127 (72.2%)	118 (66.7%)	
T4	27 (15.3%)	14 (7.9%)	
**N stage**			0.006
N0	92 (52.3%)	118 (66.7%)	
N1	44 (25.0%)	39 (22.0%)	
N2	40 (22.7%)	20 (11.3%)	
**M stage**			0.011
M0	140 (79.5%)	159 (89.8%)	
M1	36 (20.5%)	18 (10.2%)	
**Chemotherapy**			0.549
No/unknown	113 (64.2%)	120 (67.8%)	
Yes	63 (35.8%)	57 (32.2%)	
**Radiation**			
No/unknown	173 (98.3%)	172 (97.2%)	0.727
Yes	3 (1.7%)	5 (2.8%)

### Nomogram model for CC patients

**Fig. 7:**
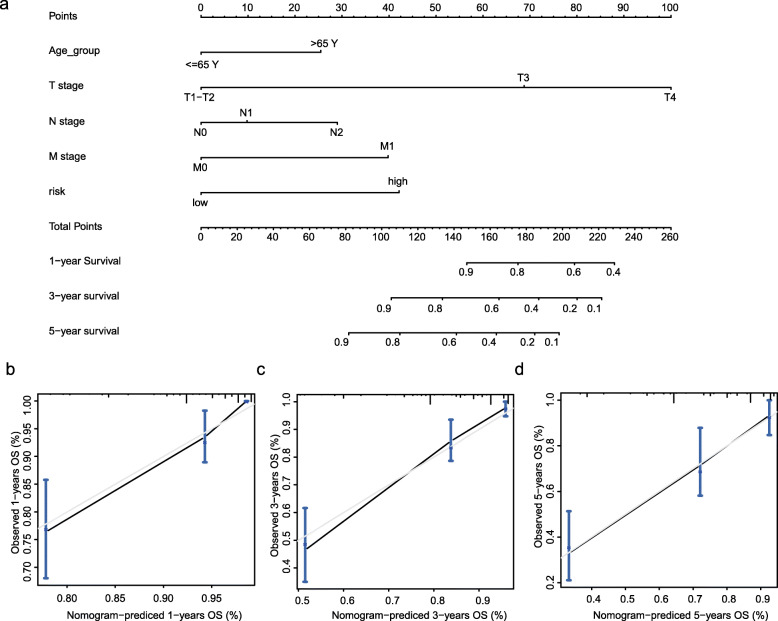
Nomogram model for colon cancer in TCGA cohort. **a** Nomogram model to predict 1-, 3-, and 5-year survival of colon cancer cases. Calibration graphs indicate that the predicted 1- (**b**), 3- (**c**), and 5- (**d**) year survival rates correspond with the actual survival rates

**Fig. 8:**
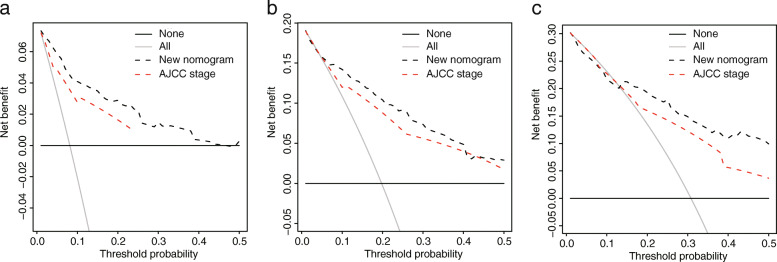
Comparison of the new nomogram model and the previous stage model. DCA showing the net benefit of our prognosis models and the previous stage model in the 1-year predicting model (**a**), 3-year predicting model (**b**), and 5-year predicting model (**c**)

The nomogram was successfully built based on multivariate models (Fig. 7a). The C-index for the nomogram was 0.838. Calibration plots demonstrated that the predicted 1-, 3-, and 5-year OS probabilities were similar to the actual observations, as shown in Fig. 7b–d. DCA demonstrated that the net benefit of our prognosis models was larger than that in the previous stage model and the other two scenarios (all screening or none-screening) in a wide range of threshold probabilities (Fig. 8a–c).

## Discussion

In this study, the expression level of 259 FRGs in CC samples and their relationship with OS were analyzed. To this end, a prognostic model containing 7 FRGs was first constructed, following which, we established a nomogram predictive model to predict OS accurately for CC patients. Functional analyses revealed that DEGs between two groups were enriched in extracellular matrix, fatty acid metabolism, and peroxisome-related pathways.

**Fig. 9:**
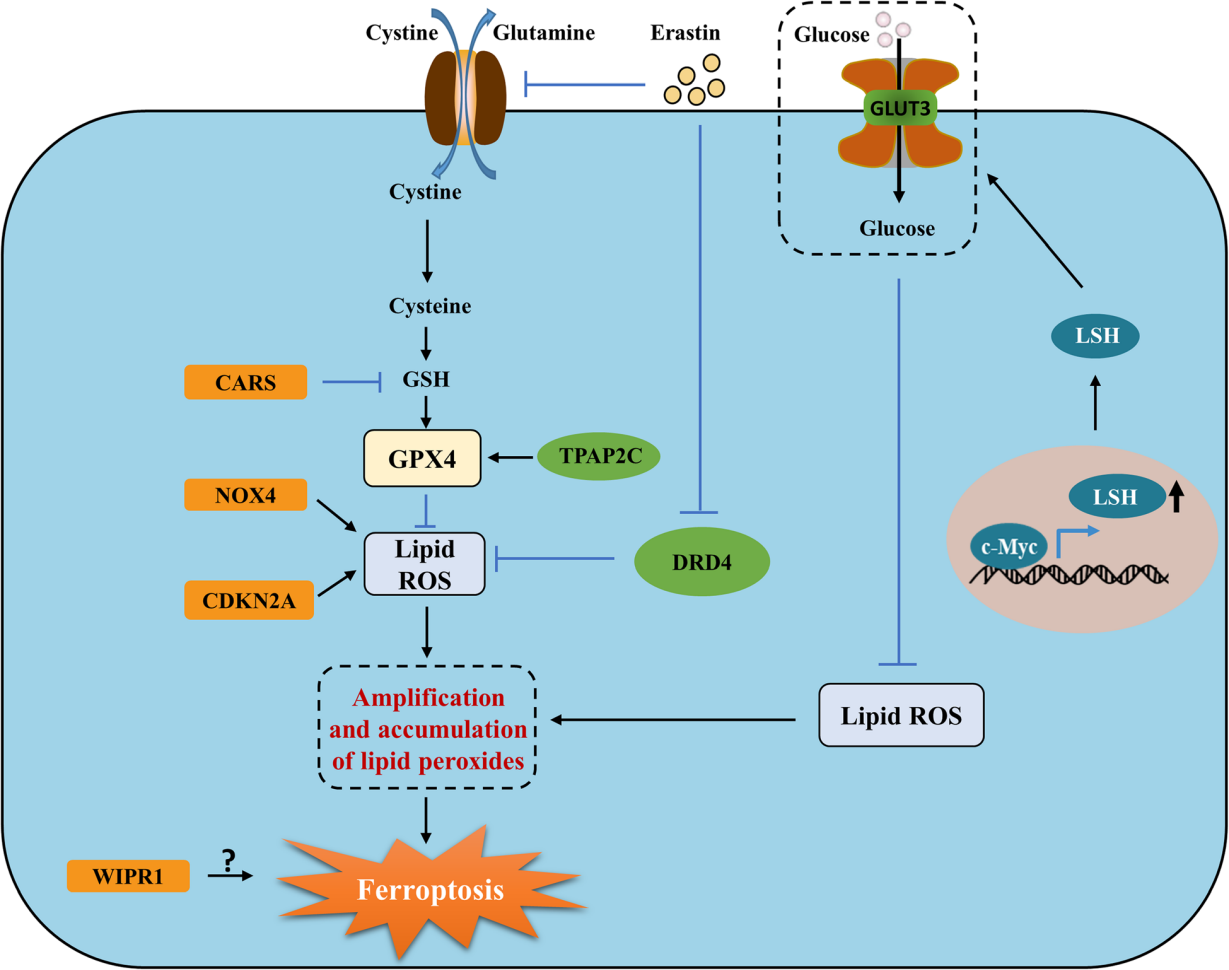
The pattern diagram of ferroptosis-related genes in colon cancer. Erastin deprives cellular cysteine, leading to GSH deletion and GPX4 inactivation. GSH is inhibited by cysteinyl-tRNA synthetase (CARS), leading to GPX4 inactivation. TPAP2C promotes the expression of GPX4 inactivation that leads to the generation of reactive oxygen species (ROS) and accumulation of lipid peroxides and final ferroptosis. NOX4, CKDN2A, and the decrease of GLUT3 and DRD4 result in accumulation of ROS, leading to ferroptosis. The mechanism of how WIPI1 regulates ferroptosis is unclear

Previous studies have shown that ferroptosis has important roles in various cancers [10, 11]. In the current study, ten prognostic DEGs were identified by analyzing the expression and prognostic effect of human ferroptosis-related genes in CC. However, given the large number of variables, overfitting is a major concern in this study and results in the inclusion of some variables with little relevance to our research question. Besides, the expression levels of different genes are not strictly independent of each other because of the many regulatory relationships among genes, which results in the multicollinearity problem [22]. The LASSO is suitable for high-dimensional data because the LASSO shrinks all regression coefficients toward zero and automatically removes many of them exactly to zero. In this case, shrinkage is desirable to prevent overfitting, and strong variable selection is desirable to obtain an interpretable prediction rule [23]. Therefore, we identified 7 FRGs from 10 prognostic FRGs to construct the risk score by using the LASSO regression. The prognostic model in this study was composed of 7 FRGs (*NOX4*, *CARS*, *WIPI1*, *CDKN2A*, *DRD4*, *SLC2A3*, and *TFAP2C*), most of which modulate the progression of ferroptosis by influencing lipid oxidation and energy metabolism. NOX4, the first identified nonphagocytic NADPH oxidase, catalyzes the generation of reactive oxygen species (ROS) from molecular oxygen to trigger ferroptosis. Meanwhile, pharmacologic inhibition of NOX4 enhances the effect of immunotherapy by conquering CAF-modulated CD8 T-cell escape [11, 24]. CARS is the rate-limiting factor for the synthesis of glutathione, a key molecule in the regulation of ferroptosis and the oxidative environment of cells [25]. Knockdown of CARS inhibits erastin-induced death, which is mediated by lipid ROS [26]. CDKN2A (also known as ARF) sensitizes cells to ROS-induced ferroptosis in a p53-independent manner, while CDKN2A depletion protects cells from ROS-induced cell death [27]. A higher expression of DRD4 has worse survival for most patients [28]. Previous studies demonstrated that erastin could induce ferroptosis through degradation of DRD4 protein, and DRD4 could inhibit the generation of ROS; it is conceivable that DRD4 might inhibit oxidative stress-induced ferroptosis [29, 30]. SLC2A3 (also known as GLUT3) encodes the glucose transporter (GLUT) protein which influences the processes of energy metabolism and is related to poor prognosis in various cancers. The downregulation of GLUT induced by the knockdown of LSH results in the generation of lipid ROS which leads to ferroptosis [31]. TFAP2C is a transcription activator that prevents ferroptosis and ferroptosis-independent modes of cell death by regulating anti-ferroptosis GPX4 expression [32]. Although experimental evidence demonstrates how WIPI1 modulates ferroptosis is lacking, RNAi screening analysis demonstrated that WIPI1 is a potential regulator of ferroptosis [33]. In summary, four of the abovementioned genes (*NOX4*, *CARS*, *WIPI1*, *CDKN2A*) promote ferroptosis and ferroptosis-independent cell death, while the remaining three genes (*TFAP2c*, *SLC2A3*, *DRD4*) have roles in protecting cells from ferroptosis. Although the expression of these genes (with the exception of *WIPI1*) are higher in colon tumor and are related to poor prognosis, whether these genes influence the survival of patients with CC by regulating ferroptosis remains unknown. The graphical abstract of FRGs in CC is showed in Fig. 9.

Seven FRGs were found to predict the prognosis of patients with CC. In addition, T stage, N stage, and M stage in the high-risk group were more advanced than those in the low-risk group, and the prognosis of the high-risk group was worse than that of the low-risk group in stage II, stage III, and stage IV as shown by subgroup analysis. These results revealed that the calculated risk score was an independent predictor of prognosis.

The nomogram was built based on multivariate models and was compared with previous AJCC stage. The results demonstrated that the new nomogram model can predict 1-, 3-, and 5-year OS better than the old staging. Thus, the new nomogram can be considered a significant model by compensating for the shortness of the previous AJCC stage prediction models for patients with CC.

Although the association between tumor progression and ferroptosis has been a key research question in recent years, the mechanism by which genes regulate colon tumor progression by influencing ferroptosis remains unclear. Based on the DEGs between the two groups, GO enrichment analysis showed that many extracellular matrix structural constituent processes were enriched. Previous studies have demonstrated that extracellular matrix detachment led to the accumulation of oxidative stress and ferroptosis [34]. Furthermore, GSEA found that the functions of DEGs were mainly enriched in fatty acid metabolism and peroxisome-related pathways. Fatty acid metabolism is dysregulated in cancer initiation and development, and the depletion of polyunsaturated fatty acids is an essential step in the process of ferroptosis [35]. Moreover, peroxisomes, which are oxidative organelles that bind with the cell membrane, are involved in ferroptosis via the synthesis of plasmalogens for lipid peroxidation [36]. These previous studies were in agreement with the results of GSEA outlined above.

There are several limitations of this study. First, there are some research biases in this prognostic model constructed from the TCGA databases. As a result, more prospective data are necessary to verify the clinical utility of the model. Second, further basic biological experiments are needed to further explore the mechanisms of FRGs in CC development. Finally, because the data in our study were acquired from the public database and the study was retrospective, there was some information that we could not collect.

In this study, we identified a novel prognostic risk score involving seven FRGs and a nomogram model in CC. This model could be used to predict the prognosis of patients with CC. Genes associated with lipid oxidation and metabolism may offer a new research direction for precise treatment strategies for CC patients.

## Supplementary Information

**Additional file 1.** Flow chart of data collection and analysis.

**Additional file 2.** The detailed results of GO analysis.

**Additional file 3.** The detailed results of GSEA.

## Data Availability

All the data and materials are available.

## Abbreviations

TCGA: The Cancer Genome Atlas
FPKM: Fragments per kilobase of exon model per million mapped fragments
CC: Colon cancer
FRGs: Ferroptosis-related genes
LASSO: Least absolute shrinkage and selection operator
DEGs: Differentially expressed genes
FDR: False discovery rate
OS: Overall survival
PCA: Principal component analysis
SE: Standard error
t-SNE: t-Distributed stochastic neighbor embedding
GO: Gene oncology
KEGG: Kyoto Encyclopedia of Genes and Genomes
GSEA: Gene set enrichment analysis
ROC: Receiver operator characteristic curve
AUC: Area under the curve
DCA: Decision-curve analysis
BP: Biological processes
CC: Cellular components
MF: Molecular functions
ROS: Reactive oxygen species
