# Supplementary figures and images for: Identification of a ferroptosis-related gene signature predictive model in colon cancer
Source: World J Surg Oncol. 2021 Apr 29;19:135. doi: 10.1186/s12957-021-02244-z (PMC8086290; doi:10.1186/s12957-021-02244-z)

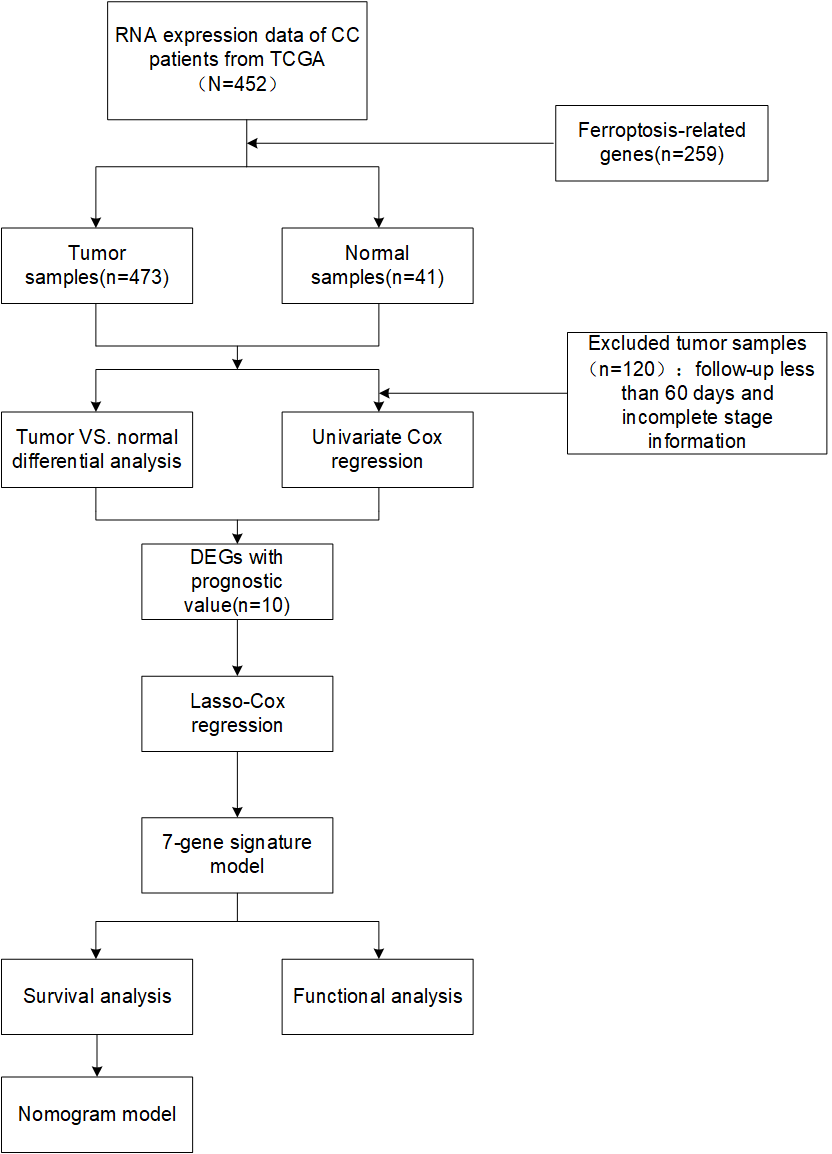

Supplement: Supplementary file 1 — Additional file 1. Flow chart of data collection and analysis. [file 12957_2021_2244_MOESM1_ESM.tif]
